# Supplementary material for: Heartfulness in Vegans, Vegetarians, and Omnivores
Source: Int J Environ Res Public Health. 2023 Mar 11;20(6):4943. doi: 10.3390/ijerph20064943 (PMC10049544; doi:10.3390/ijerph20064943)
Supplement: Supplementary file 1 [file ijerph-20-04943-s001.zip › ijerph-2237157-supplementary.pdf]

S1. Effect size calculation

Concerning the **first** hypothesis, with a small-medium effect size of f^2(V)= 0.04, an alpha-level of p = .05, and a power of *1-ß* = .80, a power analysis with G*power [1] for the MANOVA with three groups resulted in N = 225 to detect significant differences in the four aspects of heartfulness with six measurements between the three groups. For self-compassion, the negative and positive scales will be analysed separately and, as for equanimity, the even-minded state of mind and the hedonic independence will be investigated. Two MANOVA were calculated, one for the aspects gratitude, compassion and equanimity which were significantly correlated and one for the two self-compassion scales. Based on an assumed medium sized effect of f^2(V)= 0.04, an alpha-level of p = .05, and a power of *1-ß* = .80, a power analysis with G*power for each MANOVA with three groups resulted in N = 153. Besides, we will calculate two ANOVAs for the dependent variables of gratitude and compassion. Under the assumption of a small-medium effect size of f = 0.20, an alpha-level of p = .05, and a power of *1-ß* = .80, a power analysis with G*power for each ANOVA with three groups resulted in N = 246.

For the **second** hypothesis, we calculate a regression for each aspect of heartfulness. With a small-medium effect size of f^2 = .10, an alpha-level of p = .008 (due to multiple testing because of six regressions), a power of *1-ß* = .80, and thirteen possible predictors (sex, age, diet choice with three categories, reason for the diet choice, net income, importance of nutrition, education status, mindfulness practice, practice of mindfulness movement) for each dependent variable of heartfulness a power analysis for the linear regression resulted in N = 250.

References:

1. Faul, F.; Erdfelder, E.; Lang, A.-G.; Buchner, A. G*Power 3: A Flexible Statistical Power Analysis Program for the Social, Behavioral, and Biomedical Sciences. *Behav. Res. Methods* **2007**, *39*, 175–191, doi:10.3758/BF03193146.

Table S2. Summarized demographic information. Mean (*SD*) of age, diet due to environmental and ethical reasons, diet due to health reasons, active meditation and mindful movement experience (for practitioners in min per week), frequency of sexes, education state, income and importance of nutrition

	sex	education state	income	importance of nutrition [1]	age	diet due to environmental and ethical reasons	diet due to health reasons	active meditation (min/ week)	mindful movement experience (min/week)
**vegan (N = 133)**	Female: 87.2%<br>Male: 11.3%<br>Other: 1.5%	'Mittlere Reife': 8.3%<br>Apprenticeship: 7.5%<br>A-levels: 41.4%<br>Bachelor degree: 19.5%<br>Master degree: 20.3%<br>Postdoctoral: 3.0%	- 1000€: 33.1%<br>- 2000€: 34.6%<br>- 3000€: 19.5%<br>- 4000€: 8.3%<br>> 4000€: 4.5%	1: 61.7%<br>2: 36.1%<br>3: 1.5%<br>5: 0.8%	33.56 (11.414)	4.35 (.751)	3.83 (1.004)	22.431 (44.987)	44.642 (103.801)
**vege-tarian (N = 142)**	Female: 77.1%<br>Male: 22.4%<br>Other: 0.5%	Secondary school: 0.7%<br>'Mittlere Reife': 4.2%%<br>Apprenticeship: 2.1%<br>A-levels: 76.0%<br>Bachelor degree: 8.5%<br>Master degree: 7.7%<br>Postdoctoral: 0.7%	- 1000€: 72.5%<br>- 2000€: 16.2%<br>- 3000€: 8.5%<br>- 4000€: 2.1%<br>> 4000€: 0.7%	1: 40.8%<br>2: 54.2%<br>3: 4.9%	26.65 (10.530)	4.08 (.699)	3.73 (.976)	10.517 (24.239)	55.595 (214.723)
**omni-vorous (N = 144)**	Female: 58.3%<br>Male: 41.7%<br>Other: 0.0%	'Mittlere Reife': 1.4%<br>Apprenticeship: 0.7%<br>A-levels: 87.5%<br>Bachelor degree: 5.6%<br>Master degree: 3.5%<br>Postdoctoral: 1.4%	- 1000€: 85.4%<br>- 2000€: 8.3%<br>- 3000€: 2.1%<br>- 4000€: 2.8%<br>> 4000€: 1.4%	1: 22.9%<br>2: 62.5%<br>3: 13.9%<br>4: 0.7%	25.20 (10.906)	3.28 (.964)	3.76 (.853)	7.587 (31.649)	11.592 (35.658)
**total (N = 419)**	Female: 77.1%<br>Male: 22.4%<br>Other: 0.5%	Secondary school: 0.2%<br>'Mittlere Reife': 4.5%<br>Apprenticeship: 3.3%<br>A-levels: 69.0%<br>Bachelor degree: 11.0%<br>Master degree: 10.3%<br>Postdoctoral: 1.7%	- 1000€: 64.4%<br>- 2000€: 19.3%<br>- 3000€: 9.8%<br>- 4000€: 4.3%<br>> 4000€: 2.1%	1: 41.3%<br>2: 51.3%<br>3: 6.9%<br>4: 0.2%<br>5: 0.2%	28.35 (11.502)	3.89 (.933)	3.77 (.943)	13.292 (34.934)	36.996 (140.529)

Annotations: [1]categories: 1=very important, 2=important, 3=neutral, 4=not important, 5=not important at all.
Mittlere Reife = school leaving certificate after 10 years of school.

Table S3a. Correlations between the aspects of self-compassion, compassion, equanimity, and gratitude in vegans.

Variable	2	3	4	5	6
1. SC-pos	-.525**	.458**	.194*	.131	.491**
2. SC-neg	-	.058	.194*	.337**	-.059
3. Com		-	.341**	.380**	.586**
4. E-esm			-	.411**	.290**
5. E-hi				-	.310**
6. Gra					-

Table S3b. Correlations between the aspects of self-compassion, compassion, equanimity, and gratitude in vegetarians.

Variable	2	3	4	5	6
1. SC-pos	-.754**	.343**	.057	.054	.389**
2. SC-neg	-	-.157	.100	.050	-.215*
3. Com		-	-.006	.321**	.348**
4. E-esm			-	.179*	.213*
5. E-hi				-	.164
6. Gra					-

Table S3c. Correlations between the aspects of self-compassion, compassion, equanimity, and gratitude in omnivores.

Variable	2	3	4	5	6
1. SC-pos	-.744**	.235**	.036	-.132	.194*
2. SC-neg	-	-.171*	.119	.303**	-.094
3. Com		-	.045	.244**	.197*
4. E-esm			-	.251**	.104
5. E-hi				-	.232**
6. Gra					-

Abbreviations: 1 = SC-pos = self-compassion positive scale, 2 = SC-neg = self-compassion negative scale, 3 = Com = compassion, 4 = E-esm = equanimity even-minded state of mind, 5 = E-hi = equanimity hedonic independence,

6 = Gra = gratitude; *: $p < = .05$, **: $p < = .01$

Table S4. Regression-analysis (Inclusion) with the criteria self-compassion positive scale and self-compassion negative scale.

	self-compassion positive scale				
variable	*b*	*SE*	β	*t*	*p*
sex	.050	.070	.034	.715	.475
age	.013	.003	.240	3.839	<.001
education state	.013	.027	.024	.501	.616
income	-.102	.042	-.158	-2.416	.016
choice of diet	-.067	.045	-.087	-1.492	.136
importance of nutrition	.134	.054	.135	2.474	.014
diet due to environmental and ethical reasons	.001	.037	.002	.037	.971
diet due to health reasons	.130	.034	.194	3.773	<.001
active meditation experience	.000	.000	.072	1.494	.136
mindful movement experience	.000	.000	-.091	-1.930	.054
	self-compassion negative scale				
variable	*b*	*SE*	β	*t*	*p*
sex	-.107	.087	-.060	-1.231	.219
age	-.018	.004	-.247	-4.287	<.001
education state	.011	.034	.016	.317	.751
income	.010	.053	.013	.192	.848
choice of diet	.094	.056	.099	1.667	.096
importance of nutrition	.038	.068	.031	.557	.578
diet due to environmental and ethical reasons	.066	.046	.080	1.437	.152
diet due to health reasons	-.084	.043	-.103	-1.947	.052
active meditation experience	.000	.000	-.080	-1.625	.105
mindful movement experience	.000	.000	.057	1.168	.244

Table S5. Regression-analysis (Inclusion) with the criteria compassion and gratitude.

	compassion				
variable	*b*	*SE*	β	*t*	*p*
sex	-.074	.034	-.106	-2.177	.030
age	-.001	.002	-.032	-.510	.610
education state	-.003	.013	-.011	-.230	.819
income	-.028	.021	-.090	-1.355	.176
choice of diet	.018	.022	.047	.793	.428
importance of nutrition	.114	.027	.236	4.258	<.001
diet due to environmental and ethical reasons	.052	.018	.160	2.904	.004
diet due to health reasons	.018	.017	.056	1.069	.285
active meditation experience	.000	.000	.029	.590	.555
mindful movement experience	.000	.000	-.044	-.924	.356

	gratitude				
variable	*b*	*SE*	β	*t*	*p*
sex	.063	.084	.036	.751	.453
age	-.003	.004	-.052	-.826	.409
education state	-.014	.033	-.021	-.444	.657
income	-.053	.051	-.068	-1.034	.302
choice of diet	.074	.055	.079	1.354	.177
importance of nutrition	.191	.066	.159	2.905	.004
diet due to environmental and ethical reasons	.144	.044	.176	3.248	.001
diet due to health reasons	.162	.042	.200	3.895	<.001
active meditation experience	.000	.000	.067	1.378	.169
mindful movement experience	.000	.000	-.025	-.525	.600

Note: sex: 1 = female, 2 = male, 3 = other, education state: 1 = no school diploma, 2 = secondary school, 3 = 'Mittlere Reife', 4 = Apprenticeship, 5 = A-levels, 6 = A-levels, 7 = Bachelor degree, 8 = Master degree, 9 = Postdoctoral; netto income: 1 = Up to 1000€, 2 = Between 1000 – 2000€, 3 = Between 2000 – 3000€, 4 = Between 3000 – 4000€, 5 = More than 4000€
